# Publisher Correction: Finding influential nodes for integration in brain networks using optimal percolation theory

**DOI:** 10.1038/s41467-018-05686-4

**Published:** 2018-08-03

**Authors:** Gino Del Ferraro, Andrea Moreno, Byungjoon Min, Flaviano Morone, Úrsula Pérez-Ramírez, Laura Pérez-Cervera, Lucas C. Parra, Andrei Holodny, Santiago Canals, Hernán A. Makse

**Affiliations:** 1 Levich Institute and Physics Department, City College of New York, New York, NY 10031 USA; 2 Instituto de Neurociencias, CSIC and UMH, 03550 San Juan de Alicante, Spain; 3 Department of Physics, Chungbuk National University, Cheongju, Chungbuk 28644 Korea; 4 Center for Biomaterials and Tissue Engineering, UPV, Valencia, 46022 Spain; 5 Biomedical Engineering, City College of New York, New York, NY 10031 USA; 6 Department of Radiology, Memorial Sloan Kettering Cancer Center, New York, NY 10065 USA

Correction to: *Nature Communications* 10.1038/s41467-018-04718-3; published online: 11 June 2018

The original version of this Article contained an error in the last sentence of the first paragraph of the Introduction, which incorrectly read ‘Correlation of brain activity is typically measured using functional magnetic resonance imaging (fMRI), and the correlation structure is often referred to as “fu’. The correct version states ‘referred to as “functional connectivity”^2–6^’ in place of ‘referred to as “fu’. This has been corrected in both the PDF and HTML versions of the Article.

