# Supplementary material for: Finding influential nodes for integration in brain networks using optimal percolation theory
Source: Nat Commun. 2018 Jun 11;9:2274. doi: 10.1038/s41467-018-04718-3 (PMC5995874; doi:10.1038/s41467-018-04718-3)
Supplement: Supplementary file 1 — Supplementary Information [file 41467_2018_4718_MOESM1_ESM.pdf]

Supplementary Information

Finding influential nodes for integration in brain networks using optimal percolation theory

Del Ferraro et al.

Supplementary Note 1 - Finding the essential nodes for integration in the brain network

In this section, we provide the heuristic algorithms used to identify influential nodes. For each algorithm, we assign the score to each node by following the described algorithms and sort the nodes according to the score.

Degree centrality. Degree centrality is the number of nearest neighbors in the network. Degree centrality is one of the simplest metric for identifying important nodes. Hubs refer to nodes in the network with large degree.

k-core and k-shell index [1–3]. k-core (KC) refers to a subset of nodes formed by iteratively removing all nodes that have degree less than k. In other words, k-core is a maximal subgraph where all nodes have at least k neighbors. k-shell index is then the largest k value of k-core that the node belongs to. To assign k-shell index for each node, we first delete all nodes with degree $k = 1$, iteratively. The removed nodes via the process belong to k-shell with $k_S = 1$. We remove next k-shell with $k_S = 2$ and we proceed to remove all the higher shells iteratively until all nodes are removed. Then, we can assign a unique k-shell index to each node in networks. It has been shown that the importance of hub nodes can be highly diminished if they are located in the periphery of the network, i.e., the low k_s shells. On the other hands, nodes in the inner k_s shells define the core of the network and correspond to the influencers in the network [1]. However, by its own definition, the nodes in the inner shells are generally high degree nodes, therefore the k-core centrality is highly correlated with the degree.

Collective Influence [4, 5]. Collective influence (CI) is designed to approximately identify the minimal set of nodes that can produce disconnected networks, based on optimal percolation network theory [4]. Mathematically, the problem can be mapped to optimal percolation and

can be solved by the minimization of the largest eigenvalue of the non-backtracking matrix of the network [4, 5]. This optimization theory was originally developed for single networks in [4] and was extended to the case of brain networks in [5] in the context of brain network of networks. The activation of nodes in the brain network was described by a state variable σ_i, which acts as an ON and OFF switch (1 and 0, respectively) to reflect the activation/inactivation state of node i. If a node is directly inactivated, then $\sigma_i = 0$. A node can also be inactivated indirectly as a result of lacking input from its inactivated neighbors in the other network, which, mathematically, is equivalent to the McCulloch-Pitts model of neuronal activation [6]:

$$\begin{aligned} \sigma_i &= 0 && \text{direct inactivation} , \\ \sigma_i &= \Theta\Bigg(\sum_{j \in \mathcal{N}(i)} \sigma_j \Bigg) && \text{otherwise.} \end{aligned} \tag{1}$$

The sum in the second equation reflects the integration of incoming activity from all nodes j that connect to node i from other networks $\mathcal{N}(i)$, and the threshold operation via the Heaviside step function Θ indicates that a minimum of incoming activity is needed for activity to propagate [6].

The collective influence (CI) score assigned to each node i in the brain network in this model is given by [5]:

$$\mathrm{CI}_\ell(i) = (k_i - 1) \sum_{j \in \partial \mathrm{Ball}(i,\ell)} (k_j - 1) + \sum_{\substack{j \in \mathcal{F}(i)\,: \\ k_j^{\mathrm{inter}} = 1}} (k_j - 1) \sum_{m \in \partial \mathrm{Ball}(j,\ell)} (k_m - 1). \tag{2}$$

Here, $k_i \equiv k_i^{\mathrm{intra}} + k_i^{\mathrm{inter}}$ is the degree, k_i^{intra} is the number of connections of node i within its network, k_i^{inter} is the number of connections to nodes in different networks in the set $\mathcal{F}(i)$, and $\partial\mathrm{Ball}(i, \ell)$ indicates the sphere of influence of node i at distance ℓ.

Technically, CI is the contribution of each node to the eigenvalue of the non-backtracking matrix, which determines the stability of the giant component [4]. CI is an optimization measure that attempts to find the smallest set of nodes that will produce the largest damage to the giant connected component of the brain network, which is analogous to minimize the largest eigenvalue of the non-backtracking matrix [4] defined on the $2M \times 2M$ edges of the network (in the case of single networks):

$$\mathcal{B}_{k\to\ell,i\to j} = \begin{cases} 1, & \text{if } j = k \text{ and } i \neq \ell\,, \\ 0, & \text{otherwise.} \end{cases} \tag{3}$$

Thus, the matrix $\mathcal{B}_{k\to\ell,i\to j}$ has non-zero entries only when $(k \to \ell, i \to j)$ form a pair of consecutive non-backtracking directed edges, i.e. $(k \to \ell, \ell \to j)$ with $k \neq j$. In this case $\mathcal{B}_{k\to\ell,\ell\to j} = 1$. The powers of the matrix $\hat{\mathcal{B}}$ give the number of non-backtracking walks of a given length between two nodes in the network [7, 8], in analogy to the powers of the adjacency matrix which count the number of paths [9].

The CI algorithm runs as follows [5]: i) at the beginning, we choose the value ℓ of the radius of the Collective Influence sphere. In our analysis of the brain network, we use the value $\ell = 2$. We find that higher values of ℓ give nearly the same results since the networks contain short paths. The value of ℓ is always smaller than the largest path in the network, and it can be optimally chosen by systematically changing it from $\ell = 1$ to the diameter of the network. We find that the optimal set of nodes is obtained when $\ell = 2$. ii) Next, CI for all nodes is computed using Eq. (2), and the node with the largest CI is inactivated. iii) Then, the CI values of the remaining active nodes are recalculated, and the next highest CI node is inactivated. iv) Step iii) is repeated until the giant active component vanishes.

Betweenness centrality [10]. Betweenness centrality (BC) measures the influence of nodes based on the shortest paths on networks. BC for each node is defined as the number of the shortest paths that pass through the node. BC identifies crucial nodes for information flow and packing transportation by definition. This centrality can capture low-degree nodes that are strategically located between large communities. For instance, imagine a node with $k = 2$ with each link connecting to a large community of tightly connected nodes. Such a low degree node will have a large BC since all the paths between nodes in the two distinct communities will necessarily pass through this bridge node.

Eigenvector centrality [11]. Eigenvector centrality (EC) is defined as the entry of the eigenvector that corresponds to the largest eigenvalue of adjacency matrix defined as

$$A_{ij} = \begin{cases} 1, & \text{if } i \text{ and } j \text{ are connected} \\ 0, & \text{otherwise.} \end{cases} \quad (4)$$

The main idea of EC is that the influence of nodes is determined by the importance of its neighbors. Therefore, neighbors with high-scoring eigenvector centrality more contribute to the score of the node. PageRank is also a variant of EC. It has been proved in [12] that the use of the largest eigenvalue of the adjacency matrix can lead to a localization of the influence in the hubs. Thus the EC centrality is highly correlated with the high degree and contains similar information about the influencers. This localization problem is solved by replacing the adjacency matrix in the centrality by the non-backtracking matrix Eq. (3).

Closeness centrality [13]. Closeness centrality (CC) is defined as the inverse of the average distance of shortest paths between the node with all other nodes in the network. The higher closeness is, the closer it is to all other nodes in average. In practice, closeness play an important role in transportation since nodes with higher CC can disseminate information efficiently to the whole connected network via shortest paths. This centrality is mainly determined by the degree since hubs will naturally be closers to other nodes in the networks, thus, it is considered as one of the hub-centric centralities.

Supplementary Note 2 - Experimental Design and Long-term potentiation experiments

The brain network is based on long-term potentiation (LTP) experiments. LTP is a synaptic strength modification protocol that leads to changes in neuronal networks, and is believed to be one of the key mechanisms by which the brain undergoes memory processes (acquisition, consolidation, and extinction) [14–20]. It refers to the enhancement of synaptic transmission efficacy in specific neuronal connections. This mechanism has been observed to occur under natural learning conditions, yet, experimental manipulation of synaptic transmission has allowed deciphering many of its characteristics, dissecting the synaptic plasticity process from other on-going processes during memory formation. In the present work, we use experimental LTP induction in the rat hippocampus to provide an experimental model of controlled long-

range functional connectivity reorganization.

All experiments were approved by the Spanish authorities (IN-CSIC), CCNY Institutional Animal Care and Use Committee Review of Research Protocol No. 980, and were performed in accordance with Spanish (law 32/2007) and European regulations (EU directive 86/609, EU decree 2001-486). The data used in this study can be found at: http://kcorelab.org. Details of the experiments are explained in the next sections.

Subjects

A total of 37 Sprague-Dawley male rats, weighing between 250-350 g, were used in these experiments. From these, 29 animals were conserved for data analysis (6 controls for the LTP network generation in baseline conditions, 4 for NAc inactivation with DREADDs, 5 for PFC inactivation with DREADDs, 4 for Hippocampal inactivation with DREADDs, 5 for Hippocampal inactivation with TTX, 2 for S1 inactivation with DREADDs, and 3 for S1 inactivation with TTX. A total of five animals were discarded due to surgery complications or poor quality of MR images, and additional three because of leak of viral particles to the neocortex in the NAc inactivation experiments. Animals were purchased from Janvier Labs (France) and maintained under a 12/12 h light/dark cycle (lights on 07:00-19:00 h) at room temperature (22±2 C). Food and water were provided ad libitum. Rats were housed in groups (4-5 animals per cage) and adapted to these conditions for at least 7 days before any manipulation.

Surgery and electrode implantation

The animals are anesthetized briefly with isoflurane (3-4 % isoflurane in 0.8 L/min O2 flow) and then injected intraperitoneally with urethane (1.3 g/kg). After 60 minutes, the main reflexes disappearance is tested and, if necessary, a second dose of urethane is injected (1/5 of the initial dose) as reinforcement. When reflexes disappear the surgery starts. During the complete procedure animals are maintained with constant temperature (37.0-37.5 C) with a water pad. Vital constants (pulse and breath distension, heart and breath rate, and oxygen saturation) are

monitored using a paw-clip pulse oximeter (MouseOx Plus, Starr Life Sciences, Oakmont, US). A constant flow of O_2 (0.8 L/min) is supplied through a mask.

The anesthetized animal is placed in a stereotaxic frame (Narishige, Japan) and a local anesthetic is injected subcutaneously in the incision points (0.2 mL of bupivacaine). The skin is opened and retracted with suture thread hold to haemostat clamps to expose the bone surface. Special care is taken to remove all traces of blood from the skull and mussel that would decrease MRI data quality due to susceptibility artefacts. Care during surgery is maximized to prevent even minor spontaneous bleeding throughout the MRI session which would also distort the BOLD (blood oxygenation level dependent) signal. Trephine holes are made by hand with a manual driller (2 mm diameter) in the target coordinates and the dura is pinched with a curved needle at the incision points to allow the penetration of the electrodes.

A bipolar stimulation electrode made of twisted platinum-iridium wires (Teflon coated, 0.025 mm diameter, WPI, USA) is inserted in the perforant pathway, a bundle of axonal fibers that represents the principal input of information to the hippocampus (AP 0.0 mm from lambda; ML 4.1 mm from lambda; DV 2.1-2.5 mm from brain surface). A recording multichannel electrode (multichannel recording electrode, 32 channels, model A1x32-6mm-100-177, NeuroNexus, Ann Arbor, Michigan, USA) is lowered in the ipsilateral dorsal hippocampus (AP 3.5 mm from bregma, ML 2.5 mm from bregma, DV 3.5 mm from brain surface). Electrophysiological recordings are made in order to precisely position the stimulating electrode in its optimal location based on the evoked potential recorded in the hippocampus. Once in place, the multichannel recording electrode is replaced by a single channel recording probe (MRI compatible) in the dentate gyrus of the ipsilateral dorsal hippocampus. Both stimulation and recording electrodes are implanted in the brain with acrylic dental cement (SuperBond, Sun Medical, Japan) and bone cement (Palacos, Heraheus Medical GmbH, Germany) and the animal is then transported into the MRI facility.

Electrophysiological recordings

A single pulse stimulation protocol (100 μs bipolar pulse, delivered at a 0.05 Hz rate) is recorded before and after LTP induction to assess synaptic potentiation (Fig. 1a). To this end, an Input-Output curve is obtained at different stimulation intensities (50, 100, 200, 400, 800, 1000, and 1200 μA) while recording the evoked field potentials in the dentate gyrus. After filtering (0.1 Hz – 3 kHz) and amplification, the electrophysiological signals are digitized (20 kHz acquisition rate) and stored in a personal computer for offline processing with Spike2. The population spike (PS) in the hilus of the DG is measured as the amplitude from the precedent positive crest and the negative peak, and the excitatory postsynaptic potential (EPSP) is measured as the maximal slope of the raising potential preceding the PS.

fMRI measurements

Imaging experiments are carried out in a 7 Tesla scanner with a 30 cm bore diameter (Biospec 70/30v, Bruker Medical, Ettlingen, Germany). Acquisition is performed in 15 coronal slices using a GE-EPI sequence applying the following parameters: FOV= 25.25 mm; slice thickness= 1 mm; matrix= 96 $\times$ 96; segments= 1; FA, 608; TE= 15 ms; TR =2000 ms. This provides a resolution of the raw images of 0.26$\times$0.26$\times$1 mm.

Additionally, T2 weighted anatomical images are collected using a rapid acquisition relaxation enhanced sequence (RARE): FOV= 25.25 mm; 15 slices; slice thickness= 1 mm; matrix= 192$\times$192; TEeff= 56 ms; TR= 2 s; RARE factor= 8. A 1H rat brain receive-only phase array coil with integrated combiner and preamplifier, and no tune/no match, is employed in combination with the actively detuned transmit-only resonator (Bruker BioSpin MRI GmbH, Germany).

Once in the MRI scanner, the anesthetized animal is constantly supplied with a 0.6-0.8 l/min O2 and heated with a water-bath system to keep a constant temperature (37 $\pm$ 0.5 C). Physiological constants are measured as before using a paw-clip pulse oximeter (MouseOx Plus, Starr Life Sciences, Oakmont, US) equipped with a MRI compatible cable. Functional MR images are acquired before (Pre condition) and after LTP induction (POST condition) using a

low-frequency 10 Hz stimulation protocol that activates the hippocampal formation without altering synaptic plasticity, as shown before [21–24]. This stimulation consists of a block design protocol as follows (see Fig. 1d): ON periods lasting 4 s of 40 pulses train, each composed of a 10 Hz stimulation train at 800 μA. We follow the ON period by OFF period with no stimulation for 26 s. This ON/OFF sequence is repeated 10 times, for a total of 300 s.

LTP is induced inside the MRI scanner using a high frequency stimulation (HFS) protocol, consisting of 6 bursts of 8 pulses each delivered at 250 Hz, with bursts repeated 6 times with a 2 minute separation between them. The total duration of the protocol is 960 s. MR images are not acquired during LTP induction. Three hours after induction, the same low-frequency stimulation protocol as used for the PRE-LTP condition (10 Hz) is used and fMRI acquisition is performed to record the consequences of synaptic potentiation on functional connectivity.

Functional MR images are preprocessed separately using FSL 5.1 M [25, 26] and AFNI [27, 28] tools. First, the images are converted from Bruker to NIfTI format. Then, motion is corrected by aligning each volume to the mean image volume [29], slice timing correction is applied, and the brain is extracted [30]. The next step is to obtain the transformation matrix to register the functional images to a rat brain T2-weighted MRI template [31]. This registration Mark [29, 32] is performed in two steps: 1) functional images are aligned to anatomical images using a rigid-body transformation and 2) anatomical images are affine-registered to the standard template. Both matrices are concatenated but not applied to the functional images, which remained in their native space. The inverse transformation is used to bring the regions of interest (i.e hippocampus, prefrontal cortex, nucleus accumbens and the venous sinus) from the Paxinos and Watson rat brain atlas [33] to the functional space. The venous sinus is removed from the images. Afterwards, spatial smoothing using a 2-mm FWHM (full width at half maximum) Gaussian kernel is applied, followed by mean-based intensity normalization to obtain a global 4D mean of 10,000. Subsequently, linear and quadratic trends, global signal and six motion parameters (three translations plus three rotations) are regressed out. Finally, the time series are bandpass temporally filtered [0.01-0.1] Hz via Fast Fourier Transform. After this process a BOLD signal as a function of time, $x_i(t)$, is output for every voxel i in the brain. This signal is

the basis for the construction of the brain network model as we explain next.

Supplementary Note 3 - Method to construct the LTP brain network

After the BOLD signal has been obtained for every voxel in the brain, we construct the brain network model via the following procedure: (1) Identification of statistically significant activated voxels (activation map) $\to$ (2) Calculation of correlation C_{ij} between all pair of voxels in the activation map $\to$ (3) Identification of brain modules through clustering algorithms $\to$ (4) Inference of interactions J_{ij} between pairs of voxels using graphical-lasso $\to$ (5) Determination of essential influential nodes using the CI algorithm from optimal percolation theory.

Activation map

We first determine which brain voxels are activated by the low-frequency stimulation protocol using the FEAT analysis tool in FSL (https://fsl.fmrib.ox.ac.uk/fsl/fslwiki/FEAT). The regression assumes for the explanatory variable the block-design of the low-frequency stimulation as described above. After the general linear model (GLM) analysis, the Z statistic map is thresholded and cluster corrected (cluster Z threshold $= 2.3$). Figure 1f shows the activation map for a single animal in the POST-LTP condition. In Supplementary Figure 1a we show the same activation map but averaged over the six animals. This map represents voxels that are activated in the POST-LTP state in at least 2 out of 6 animals with $p < 0.001$ (determined after co-registering the fMRI recordings to a common anatomical rat brain atlas of Paxinos and Watson [33]). Supplementary Figure 1b shows the anatomical areas corresponding to the HC, PFC, and NAc. Comparison between both images indicates that voxels in these three areas are activated after the LTP induction. These activated areas form the basic voxels used as "nodes" in the subsequent calculation of the brain network model.

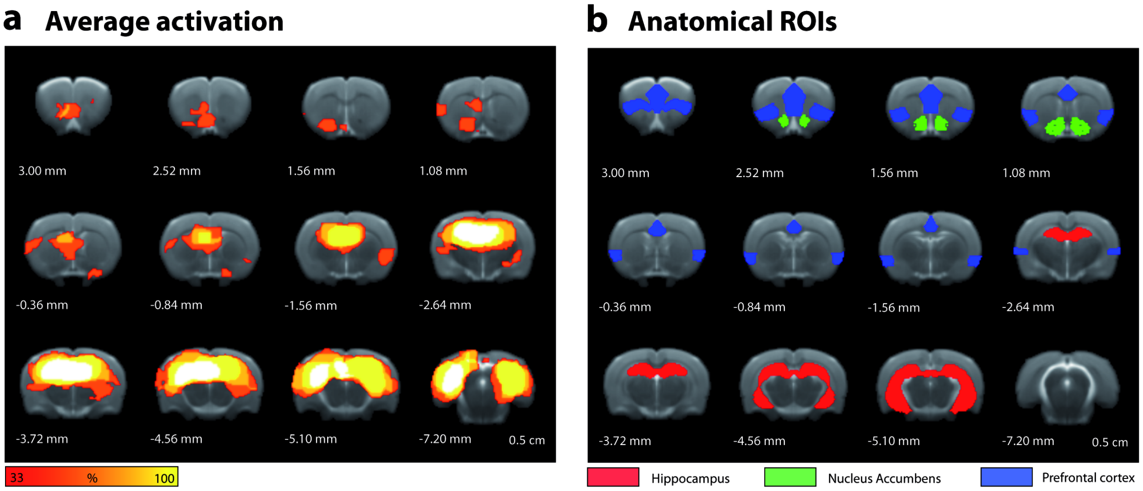

Supplementary Figure 1: Activation map and anatomical areas of interest. **a,** Group (n=6) average activation map after LTP induction. This map represents voxels that are activated three hours after LTP stimulation (POST condition) in at least 33% of the animals with $p < 0.01$ (see Supplementary Note 3). Note activation in the hippocampus (HC), prefrontal cortex (PFC) and nucleus accumbens (NAc). Numbers indicate distance from bregma. **b,** Anatomical map defining the three main areas of study: HC, PFC and NAc.

Construction of memory networks

In order to construct the brain network we first compute the correlation coefficients or sample covariance C_{ij} of the BOLD signal between voxels i and j in the activation map, often referred to as "functional connectivity":

$$C_{ij} = \frac{\langle x_i x_j \rangle - \langle x_i \rangle \langle x_j \rangle}{\sqrt{(\langle x_i^2 \rangle - \langle x_i \rangle^2)(\langle x_j^2 \rangle - \langle x_j \rangle^2)}}, \tag{5}$$

where $x_i(t)$ is the BOLD signal of voxel i as a function of time t and $\langle \cdot \rangle$ represents the temporal average over the recording period. Correlations are computed separately for each animal for all voxels that showed significant activation in at least 2 animals (activation maps were co-registered to a standard atlas, but correlation is computed in the original space to avoid in-

troducing spurious correlations due to resampling).

In the animal original space, the BOLD signal is measured at a resolution of 0.26×0.26×1 mm. Another source of spurious correlations might arise when applying the customary spatial smoothing to the image with a Gaussian kernel, because the volume space is not isotropic. So, to avoid including spurious correlations of fMRI signals in the (x, y)-plane, we consider only every four voxels so that nodes are separated by $1.04 \times 1.04 \times 1$ mm, and are approximately isotropic in all three dimensions. Therefore, the size of the voxel, that is, each node in the brain network, is approximately 1 mm^3 and this corresponds to a single node in the network. This size is commensurate with the size of the target in the pharmacogenetic interventions. The same downsampling procedure described above is applied in all the analysis described in the text, with or without pharmacogenetic intervention. Following existing literature we model these correlations as the result of pairwise interactions between nodes [34–38].

Inference of the connections of sparse network

The pair-wise correlation modelling literature typically assumes that brain networks have sparse connectivity [35–38, 40]. We therefore construct sparse graphs by using machine learning techniques like the graphical Lasso algorithm [41]. Given normal distributed data, the log-likelihood for observing the sample covariance $\mathbf{C} = \{C_{ij}\}$, defined in Eq. (5, is given by the log of the Wishart distribution:

$$\log L(\mathbf{J}) = \log\det(\mathbf{J}) - \mathrm{Tr}(\mathbf{CJ}), \tag{6}$$

where $\mathbf{J} = \{J_{ij}\}$ is the model for the inverse covariance. These J_{ij} reflects the strength of interactions between a pair of nodes i and j. To implement the assumption of sparse interactions the Graphical Lasso algorithm assumes a Laplace prior, which results in a maximum a posteriori estimate with a L1-norm penalty term [41]:

$$\mathbf{J}^* = \underset{\mathbf{J}}{\mathrm{argmin}}[\mathrm{Tr}(\mathbf{CJ}) - \log\det(\mathbf{J}) + \lambda|\mathbf{J}|], \tag{7}$$

where $|\mathbf{J}|$ is the L1-norm of the interaction matrix and λ is the penalty parameter controlling how sparse the estimated $\mathbf{J}^*$ will be. A sparse interaction matrix will have many zero entries.

A non-zero entry indicates that there is a pair-wise interaction, while $J^*_{ij} = 0$ means that there is no direct interaction between i and j. We infer the sparse matrix J_{ij} fixing the λ penalization parameter in Eq. (7) as described below, for each separate animal.

Since we are interested to study the integration of a set of networks aggregated into a giant connected component, we define the brain network via a procedure involving a change in the penalty parameter λ, which tunes the sparsity of the network (see Eq. (7)). A giant component is a connected component of a given graph that contains a constant fraction of the entire graph's vertices in the thermodynamic of an infinite system size. As λ is changed from a high value to a low value, a series of networks emerge to form the giant connected component of brain network in a procedure that we explain below. Higher values of λ penalize almost all of the links and therefore the brain network is disconnected. As we reduce the values of λ in Eq. (7), more links appear and the brain network transforms into a giant connected components of nodes (inside this component there is a path connecting every pair of nodes). For a finite graph, we consider the giant component as the largest connected component in the graph and study the behaviour of its relative size $G_{\rm bond}$ as a function of λ. In these plots, $G_{\rm bond}$ represents the ratio of nodes belonging to the largest connected component to the total number of nodes in the brain network. The suffix bond refers to the fact that this process builds the brain network via a process analogous to bond percolation (see below) [42–45]. Thus, we use the 'bond' denomination of this giant connected component since it is constructed by adding links to the network by reducing the penalty parameter λ. Indeed this process is analogous to bond-percolation and attempts to solve the problem of choosing the thresholding or penalty parameter that defines the binary network from the weighted covariance matrix by using the concept of the emergence of the giant connected component. That is, following [46] we choose the penalty in such a way that the resulting network is at the point of emergence of the connected components that connect each cluster HC, PFC and NAc in turn. This process results into a sparse, yet, connected network and it follows the idea that the most important feature of the network that we want to capture in our study is the long-range connectivity and integration of the different components into a unitary network. Thus, we whole analytical procedure starts by findings the sparse connected

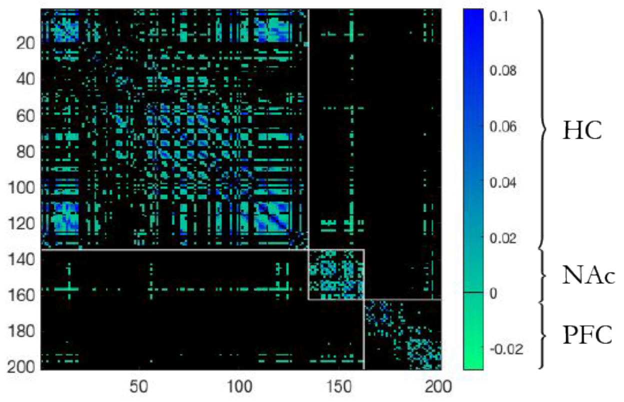

Supplementary Figure 2: For the same representative animal of Fig. 1f and 1h: Adjacency matrix of the resulting brain network, obtained by bond-percolation using the penalty parameter λ in the graphical lasso algorithm as described in Supplementary Note 3. Nodes are ordered according to their membership to one of the anatomical clusters: HC, PFC and NAc. From above to below, the first module corresponds to the HC, the second to the PFC, the third to the NAc.

network of HC-PFC-NAc via graphical-lasso and bond percolation of the penalty parameter to then apply the optimal percolation method via the collective influence algorithm to find the essential nodes for inactivation. We explain this procedure next.

In a percolation problem one monitors the size of (fraction of nodes belonging to) the giant connected component $G_{\rm bond}$ as a function of the driving external parameter. In the present case, we first apply the graphical lasso for a given λ and obtain the inferred matrix J_{ij} from C_{ij}. We binarize this matrix and construct a network by considering a link if $|J_{ij}|$ is above a given small resolution threshold, as it is customary in the graphical lasso algorithm. We then monitor the giant component of this network for a given λ versus the penalty parameter λ and we search for the appearance of the giant component as λ is decreased from a large value. The process of constructing the network by decreasing λ adds links to an initially empty network as in bond percolation. We fix the penalty parameter λ, which tunes the sparsity of the network, as the highest value at which the giant component of the network appears between each cluster, in turn. i.e. such that all nodes in the three clusters HC-PFC-NAc are connected through a path.

In other words, the final network is the sparsest architecture that yet has one connected giant component which includes nodes from the three clusters. The connectivity matrix is obtained by binarizing the obtained J_{ij} from the graphical lasso at a given λ by considering a link when J_{ij} is non-zero with a given small resolution. The resulting connectivity matrix from J_{ij} is shown in Supplementary Fig. 2, for the same representative animal used in Fig. 1f and 1h. From this matrix we identify the three anatomical components HC, PFC and NAc and the links inside the clusters or strong or intra-links and the links across the clusters, the weak or inter-links [46].

Supplementary Note 4 - Measure of average maps of centralities in the brain network

For all nodes in the brain network we compute the score of each centrality for each experimental animal. We then rank all the nodes from high to low score. We then 'attack' the brain network following each ranking for each centrality from hubs, CI, KC, EC, CC and BC. We monitor the size of the giant component as we remove a fraction of influential nodes q following each strategy and for each network corresponding to each of the six animals [4, 5]. Supplementary Figure 3 shows the results. We see how the strategy following CI destroys the giant component with the smallest number of nodes. For each strategy, we extract the set of most influential nodes, the essential nodes according to each strategy, by considering the first nodes that reduce the size of the connected component to 5% of its original size. These are the set of essential nodes for each centrality and correspond to the ranking of top nodes according to each centrality.

Lastly, we normalize the ranking of each node using the following formula [5] to compare across strategies:

$$R(i) = \frac{r_o - r_i}{r_o - 1}, \tag{8}$$

where r_i is the ranking of node i, that is defined as the step at which it is inactivated (for example, the first node to be inactivated is assigned $r_i = 1$, the second $r_i = 2$, and so on). The quantity r_o is a baseline, which, in our analysis, we set as the ranking of the node for which the giant active component takes the value $G = 0.05$. Note that $R(i) = 1$ represents the highest node. On the other hand, if node i is not targeted by an external inactivation, then we set $R(i) = 0$. The

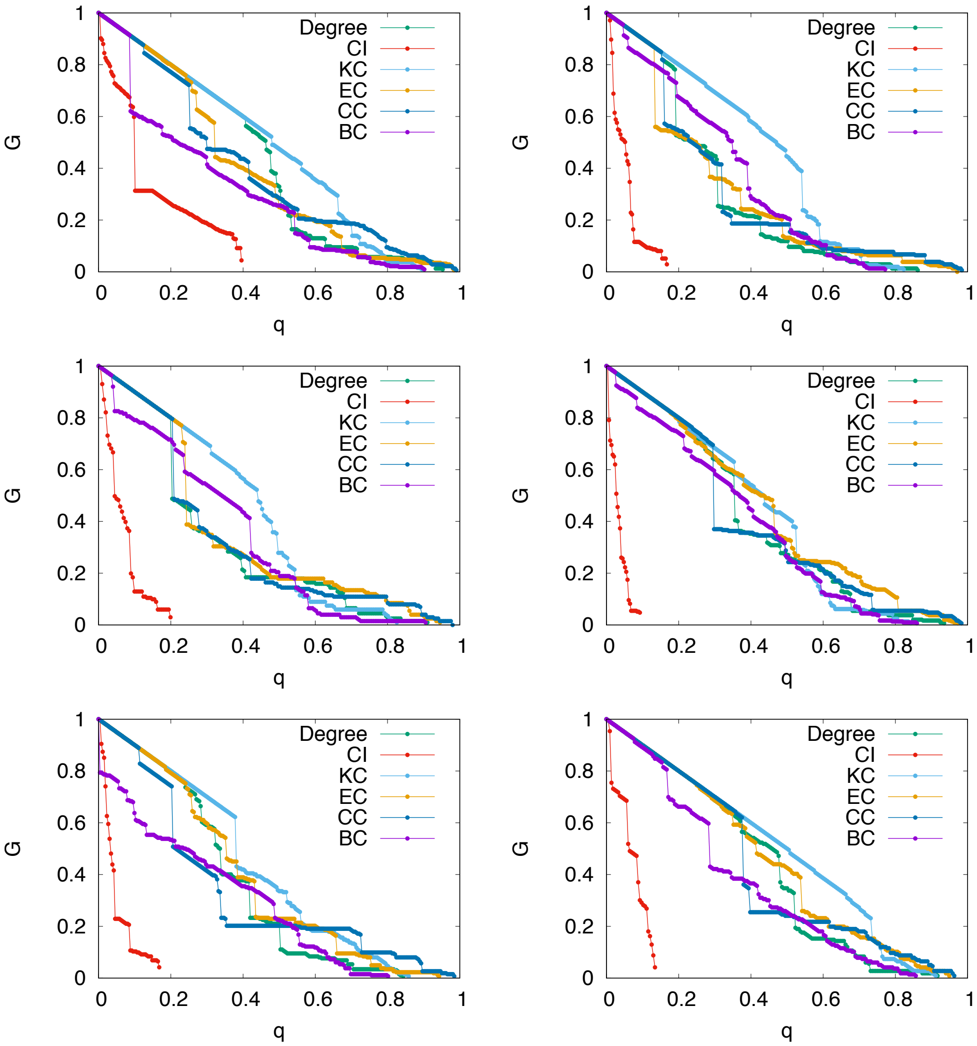

Supplementary Figure 3: Size of the giant connected component G as a function of the fraction of inactivated nodes, q for all six rats for degree, CI, KC, EC, BC, and CC. For CI, smaller number of inactivated nodes are required to disintegrate the network consistently for all six rats.

normalization in Eq. (8) allows us to properly sum over all samples to get an averaged map of the most important nodes in the brain network which allows us to compare the impact of each centrality. The results are used to generate the the hub-map in Fig. 2g and the averaged CI map in Fig. 2h, as well as all the centrality maps shown in Fig. 3.

Supplementary Note 5 - Influencers map for the resting state dynamics

In this section we present results regarding which nodes are responsible for integration during resting state dynamics, as discussed in Sec. 2. The analysis of the essential nodes for integration presented in the main text, indeed, is performed on brain networks stimulated by LTP induction which, in addition to the hippocampus, produces the activation of the prefrontal cortex and of the nucleus accumbens. In the PRE-LTP condition, stimulation of the hippocampus does not recruit activation of neither the PFC nor the NAc and therefore, the relevance of these latter areas for brain integration cannot be investigated. To clarify their role in the brain network, we analyze the fMRI signal of the resting state dynamics in a PRE-LTP condition.

Since we are interested in investigating the role of the HC, the PFC and the NAc during unperturbed brain dynamics, we take into consideration the same anatomical areas, i.e. same voxels, studied to analyze the LTP-induced network. This guarantees that nodes in the resulting brain networks are the same for both the LTP-induced and the resting-state network. What changes between the two cases are the BOLD signals and, therefore, the statistical dependences between these voxels, i.e. the wiring of the resulting architecture.

The analysis is done on the same six animals presented in the main text (in that case POST-LTP), same p-value ($p < 0.001$). Each resting-state brain network is constructed similarly to the LTP-induced one, as described in Sec. 2 and in Supplementary Note 3. For each of these networks we rank the nodes according to the CI centrality measure, obtaining a CI-map for each rat. We then average the CI-score across the six animals, similarly to the LTP-induced networks, as described in Supplementary Note 4. The averaged results are shown in Supplementary Figure 4 which shows no role for the NAc as director of brain integration. High CI-score nodes, indeed, are less localized to a single brain area and are rather spread in different brain regions, mostly involving the hippocampus and the prefrontal cortex. These findings demonstrate that brain integration is related to brain dynamics. The role of the NAc as director of brain integration discussed in the main text is not simply arising because of its anatomical location in the brain but, rather, it is due to the functional re-organization stimulated by LTP-induction.

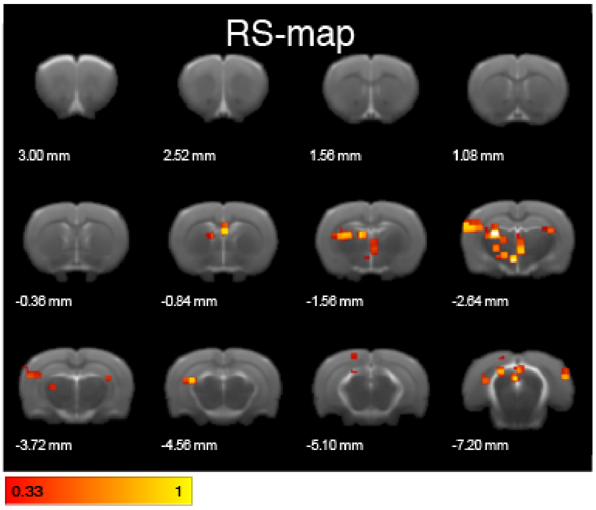

Supplementary Figure 4: Average ($n = 6$) CI-map for the resting-state brain dynamics. The map shows the CI-score (Eq. (2)) of each voxel averaged over six animals for the case of unperturbed brain PRE-LTP induction. High CI-score voxels are not localized in a single brain areas but appears spread around and mostly located between the hippocampus and the prefrontal cortex.

Supplementary Note 6 - Directed brain network analysis

The network analysis of influencers in the rodent brain presented in Sec. 2 and 2 is based on the construction of an undirected network. All biological networks are directional and so is the neural wiring in the brain. At the neuronal level, indeed, each synapse and axon has a specific direction for the flow of electric and chemical signal. A single voxel, which is the maximal spatial resolution of a fMRI scan, contains about 10^4 neurons. The information flow between two voxels can be thought as resulting from the average flow of chemical and electric signals between all the neurons in these voxels.

To date, Granger causality [47] is a useful tool to statistically test probabilistic causal and directional relations between two temporal variables and since its introduction in 1969, it has been applied in several disciplines, ranging from finance to neuroscience and biology. In this section we re-construct the same brain networks induced by LTP for the rodent brain made of the active brain areas during fMRI scans, i.e. HC, NAc and PFC, as discussed in the main text

and, in addition, we use Granger causality [47] to infer probabilistic directions of the network's links. We first start from the undirected network as discussed in Section 2 for each one of the six animals. For each connected pair of nodes in the functional network throughout an undirected link, we infer directionality of the connection by applying Granger causality to the BOLD signal of the pair of voxels. We use a confidence level $\alpha = 0.01$ and a lapse $t_l = 1$-step in the scanning time, which correspond to 2 seconds, this is the minimum temporal resolution available from the fMRI in use.

Given two voxels i and j, from their time series, we test the hypothesis i Granger-causes j and, if the hypothesis is accepted, we assign a link $i \to j$. We then test the opposite hypothesis: j Granger-causes i. If both hypothesis are accepted we add no directionality to the link $i - j$, the same in the case when none of the two hypothesis is accepted. Therefore, directionality is assigned when either i Granger-causes j ($i \to j$) or j Granger-causes i ($j \to i$).

To find which are the influencers, i.e. the integrators, in this directional network we develop an heuristic version of the Collective Influence (CI) algorithm, presented in Eq. (2) in the SI, which accounts for link directionality. Once the network is directed, each node has a given in-degree (k_i^{in}) and out-degree (k_i^{out}) and undirected links contribute to both of them. A natural generalization of the CI algorithm to the directed case is then the following:

$$\text{CI}^{\text{DIR}}{}_{\ell}(i) = (k_i^* - 1) \sum_{j \in \partial\text{Ball}^*(i,\ell)} (k_j^* - 1) + \sum_{\substack{j \in \mathcal{F}^*(i)\,: \\ k_j^{*\text{in}-\text{inter}} = 1}} (k_j^* - 1) \sum_{m \in \partial\text{Ball}^*(j,\ell)} (k_m^* - 1). \tag{9}$$

Where here, slightly differently from the undirected case, $k_i^* = k_i^{\text{in}} + k_i^{\text{out}}$ is the total degree of node i, with $k_i^{\text{in}} \equiv k_i^{\text{in}-\text{intra}} + k_i^{\text{in}-\text{inter}}$ that accounts for: the total in-links coming from nodes in the same network as i ($k_i^{\text{in}-\text{intra}}$); and in-links coming from nodes belonging to a different network than i ($k_i^{\text{in}-\text{inter}}$). Analogously, $k_i^{\text{out}} \equiv k_i^{\text{out}-\text{intra}} + k_i^{\text{out}-\text{inter}}$, with $k_i^{\text{out}-\text{intra}}$ and $k_i^{\text{out}-\text{inter}}$ having a similar meaning but for the out-degree of node i. Diversely from Eq. (2), the symbol $\partial\text{Ball}^*(i,\ell)$ indicates the *directed* sphere of influence of node i: this is the sphere of influence that can be reached with a directed path starting at node i. Whereas $j \in \mathcal{F}^*(i)$: $k_j^{*\text{in}-\text{inter}} = 1$ instead indicates the set of nodes connected to i through a *directed* interlink and which have no more interlinks with any of the other nodes in the network.

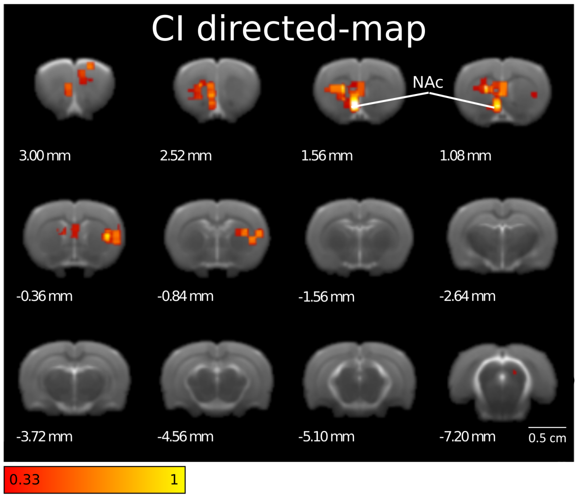

Supplementary Figure 5: Average ($n = 6$) CI-directed map. The map indicates the CI-score (Eq. (9)) of each voxel averaged over six animals for the case of directed brain network. The Nucleus Accumbens appears as the area with the highest CI averaged score and, therefore, it is identified as the main area responsible for integration.

To identify the influencers of the directed brain network, for each rat, we compute the directed CI-score according to Eq. (9), in analogy with the undirected case, for each node in the brain network. For each animal, we then rank the nodes from high to low score and we then compute an average CI-directed map similarly to what described in Supplementary Note 4. Results are shown in Supplementary Fig. 5, to be compared with results for the undirected network discussed in the main text and illustrated in Fig. 2h. Despite the fact that the networks are directed in this case, the nucleus accumbens still results to be the brain area with the highest CI-directed score and so, according to our theory, the main brain areas responsible for integration.

Supplementary Note 7 - Degree analysis of nodes responsible for integration

In this section we present a study of the degree statistics for the top CI nodes in each rodent brain network and of the top hub nodes in the same network. In particular, for each rat brain

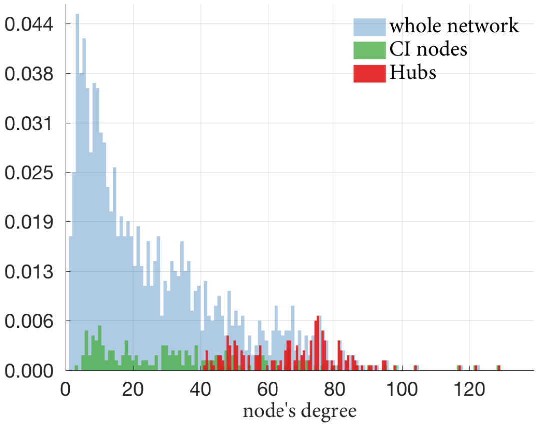

Supplementary Figure 6: Degree distribution across animals ($n = 6$) of: (blue bins) the whole brain network; (green bins) top 30 CI nodes; (red bins) top 30 hub nodes. The figure shows that the top CI nodes, which our theory identify as responsible for brain integration, are comparatively of lower degree than hub nodes in the same networks.

network, we identify the top 30 CI nodes according to equation (2) and we then determine the degree of each one of these nodes in their relative network. Analogously, for each rat, we also identify the top 30 hub nodes by using a high-degree algorithm and then determine their degree. We choose the first 30 nodes because, across animals, this is the max number of CI nodes which can be removed before the network is completely dismantled and so, the max number of nodes which can be used to compare the CI and hub degree statistics. For completeness, we also compute the degree statistics of all rodent brain networks by identifying the degree of each node in the network. In Supplementary Fig. 6 we report the corresponding degree distributions obtained from the above analyses. This figure illustrates that high CI nodes, i.e. nodes that we find responsible for integration within our theory, are comparatively of lower degree than hubs in the brain network.

Supplementary Note 8 - Pharmacogenetic (Dreadd) experiment

The fundamental goal of this experiment is to use Designer Receptors Exclusively Activated by Designer Drugs (DREADDs) technology [48, 49] to specifically inactivate nodes in the shell part of the nucleus accumbens (NAc), the contralateral hippocampus (cHC), the anterior part of the prefrontal cortex (PFC), and the somatosensory cortex (S1) and study, using fMRI and optimal percolation analysis, its impact on the functional architecture in the memory network induced by LTP. More specifically, with the aid of adenoviral vectors, we directed the expression of a Gi-DREADD (hM4Di) protein into the target regions which, under intra-peritoneal administration of the otherwise inert ligand clozapine-N-oxide (CNO), activates the receptor inducing neuronal silencing and blocking those regions output. Details are provided below.

Subjects

A total of 15 Sprague-Dawley male rats, weighing between 260-280 g, were used in this experiment. From these, three animals were not considered in the analysis due to absence or poor DREADD expression in the post-mortem validation. As before, animals were purchased from Janvier Labs (France) and maintained under a 12/12 h light/dark cycle (lights on 07:00-19:00 h) at room temperature (22$\pm$2 C). Food and water were provided ad libitum. Rats were housed in groups (4 animals per cage) and adapted to these conditions for at least 7 days before any manipulation.

Viral constructs and injection procedures

A mixture of two viruses is used to express hM4Di in the NAc. The first virus (AAV5-hSyn-GFP-Cre) drives the expression of Cre under the control of Synapsin (hSyn) in neurons and provides amplification of the Cre-dependent DREADD construct. The second virus (AAV5-hSyn-DIO-hM4D(Gi)-mCherry) expresses the inhibitory DREADD in Cre positive neurons. Both viruses are mixed 1:1 and 0.25 μL are injected stereotaxically in the shell portion of the

NAc. For this, isoflurane anesthetized animals (4 % induction and 2.5 % for maintenance in 0.8 L/min O_2) are fixed in an stereotaxic frame, as described above, and bilateral craniotomies opened over the NAc (from bregma, AP 2.5 mm, ML 1.3 mm, and DV 7 mm), the PFC (from bregma AP -3.2 mm, ML 0.5 mm, and DV 2.0 and 3.8 mm), the contralateral Hippocampus (from bregma, AP -3.5 mm, ML 2.6 mm, and DV 3.2 mm), and the somatosensory cortex S1 (from bregma, AP 0.8 mm, ML 3.6 mm, and DV 1.4 mm). Injections are performed using silica cannula (GC22-20, 22 gauge internal cannula, WPI, USA) coupled to an infusion pump (SP200IZ Syringe pump, WPI, USA) through polyethylene tubing. The cannula is lowered slowly in the tissue to the final stereotaxic coordinate, stays in place 10 min before infusion starts, and 10 min more before retraction. Retraction is done slowly to prevent sucking the injected solution. At the end of the procedure, both craniotomies are covered with small amounts of bone cement (Palacos, Heraheus Medical GmbH, Germany), and the skin sutured. After the surgery animals receive analgesics (buprenorphine 0.3 mg, Buprex, Reckitt Benckiser Healthcare, UK) and antibiotics (enrofloxacine 3 %, Syvaquinol 25, Syva, Spain) during 3-5 days.

DREADD fMRI procedures

We wait 4 to 6 weeks after the injection of the viruses to allow proper expression of the DREADD proteins in the NAc neurons. The experimental procedures for electrode implantation and fMRI data acquisition are the same as explained above for the LTP experiment. In addition, animals in this experiment are intraperitoneally cannulated for CNO administration inside the magnet. After baseline fMRI acquisition is completed (corresponding to the PRE-LTP, PRE-CNO condition), CNO is administered i.p (1 mg/Kg, 10 mL/Kg) and 30 min later a first set of functional images is acquired during low frequency stimulation (PRE-LTP, POST-CNO condition). After that, and still under the effect of CNO (which last more than 10 h, [50]), LTP is induced as before and 1h later a new set of functional images is acquired (POST-LTP, POST-CNO condition).

Histology

At the end of each experiment, rats are perfused intracardially with 100 mL of 1% phosphate-buffered saline (PBS) solution and 100 mL of ice-cold 4% paraformaldehyde (PFA). Brains are kept for 24h on 4% PFA post-fixation at 4 C and cut in a fixed material vibratome in 50 μm thick slices. Slices are then stained with 4',6-diamidino-2-phenylindole (DAPI) for photography under a fluorescence microscope. Expression of hM4Di in the NAc is validated by GFP fluorescence in the neurons.

Supplementary Note 9 - Pharmacologic (TTX) inactivation experiments

In this experiment, we used an acute infusion of the voltage-dependent sodium channel blocker Tetrodotoxin (TTX), to strongly inactivate nodes in targeted regions and study, using fMRI and optimal percolation analysis, its impact on the functional architecture in the memory brain network induced by LTP.

Subjects

A total of 8 Sprague-Dawley male rats, weighing between 250-300 g, were used in this experiment, 4 for Hippocampal inactivation and 3 for S1 inactivation. As before, animals were purchased from Janvier Labs (France) and maintained under a 12/12 h light/dark cycle (lights on 07:00-19:00 h) at room temperature (22 $\pm$ 2 C). Food and water were provided ad libitum. Rats were housed in groups (4 animals per cage) and adapted to these conditions for at least 7 days before any manipulation.

Drug and injection procedures

Urethane anesthetized animals are fixed in a stereotaxic frame, as described above, and craniotomies are opened bilaterally over the Hippocampus (from bregma, AP -3.5 mm, ML 2.6 mm,

and DV 3.2 mm), or the somatosensory cortex S1 (from bregma, AP 0.8 mm, ML 3.6 mm, and DV 1.4 mm). Injections are performed using silica cannula (GC22-20, 22-gauge internal cannula, WPI, USA) coupled to an infusion pump (SP200IZ Syringe pump, WPI, USA) through polyethylene tubing. The cannula is lowered slowly in the tissue to the final stereotaxic coordinate, stays in place 10 min before infusion starts, and 10 min more before retraction. $0,5\mu$L of TTX (100 μM in ACSF) are infused in the target region. Retraction is done slowly to prevent sucking the injected solution. Two multichannel recording electrodes are inserted in the ipsilateral and contralateral Hippocampus to account for the induced TTX inactivation and the successful induction of LTP. After TTX is infused as described above, field potentials in the contralateral Hippocampus are abolished, whilst field potentials in the ipsilateral Hippocampus remain intact (not shown). After that, LTP induction and fMRI procedures proceed as described in the main text and Supplementary Note 2.

Supplementary References

[1] Kitsak, M., Gallos, L. K., Havlin, S., Liljeros, F., Muchnik, L., Stanley, H. E. & Makse, H. A., Identification of influential spreaders in complex networks, *Nature Phys.*, **6**, 888-893, (2010).

[2] Hagmann, P., Cammoun, L., Gigandet, X., Meuli, R., Honey, C. J., Wedeen, V. J. and Sporns, O., Mapping the structural core of human cerebral cortex, PLoS Biol, **6**, e159, (2008).

[3] Seidman, S. B., Network structure and minimum degree. *Soc. Networks* **5**, 269-287, (1983).

[4] Morone, F. & Makse, H. A., Influence maximization in complex networks through optimal percolation, *Nature*, **524**, 65-68, (2015).

[5] Morone, F., Roth, K., Min, B., Stanley, H. E. & Makse, H. A., Model of brain activation predicts the neural collective influence map of the human brain, *Proc. Natl Acad. Sci. USA*, **114**, 3849-3854, (2017).

[6] McCulloch, W. & Pitts, W., A logical calculus of the ideas immanent in nervous activity, *B. of Math. Biophys.* **5**, 115-133, (1943).

[7] Hashimoto, K., Zeta functions of finite graphs and representations of p-adic groups. *Adv. Stud. Pure Math.*, **15**, 211-280, (1989).

[8] Angel, O., Friedman, J. & Hoory, S., The non-backtracking spectrum of the universal cover of a graph, *Trans. Amer. Math. Soc.*, **367**, 4287-4318, (2015).

[9] Newman, M. E. J., *Networks: An Introduction*, (Oxford University Press, Oxford, 2010).

[10] Freeman, L. C., A set of measures of centrality based on betweenness, *Sociometry*, **40**, 35-41, (1977).

[11] Straffin, P. D., Linear algebra in geography: eigenvectors of networks, *Mathematics Magazine*, **53**, 269-276, (1980).

[12] Martin, T., Zhang, X. & Newman, M. E. J., Localization and centrality in networks, *Phys. Rev. E*, **90**, 052808, (2014).

[13] Bavelas, A., Communication patterns in tasks oriented groups, *J. Acoust. Soc. Am.*, **22**, 271-282,

(1950).

[14] Bliss, T. V. P., Collingridge, G. L. & Morris, R. in *The Hippocampus book*, Andersen, P., Morris, R., Amaral, R., Bliss, T. V. P. & O'Keefe, J. (Oxford University Press, Oxford, 2007).

[15] Lynch, M. A., Long-term potentiation and memory, *Physiol. Rev.*, **84**, 87-136, (2004).

[16] Bliss, T. V. & Lomo, T., Long-lasting potentiation of synaptic transmission in the dentate area of the anaesthetized rabbit following stimulation of the perforant path, *J. Physiol*, **232**, 331-356, (1973).

[17] Bliss, T. V. & Collingridge, G. L., A synaptic model of memory: long-term potentiation in the hippocampus, *Nature*, **361**, 31-39, (1993).

[18] Martin, S. J., Grimwood, P. D. & Morris, R. G. M., Synaptic plasticity and memory: An Evaluation of the Hypothesis, *Annu. Rev. Neurosci.*, **23**, 649-711, (2000).

[19] Morris, R. G., Episodic-like memory in animals: psychological criteria, neural mechanisms and the value of episodic-like tasks to investigate animal models of neurodegenerative disease, *Philos. Trans. R. Soc. Lond. B Biol. Sci.*, **356**, 1453-1465, (2001).

[20] Squire, L. R., Mechanisms of memory, *Science*, **232**, 1612-1619, (1986).

[21] Canals, S., Beyerlein, M., Merkle, H. & Logothetis, N. K., Functional MRI evidence for LTP-induced neural network reorganization, *Curr. Bio.*, **19**, 398-403, (2009).

[22] Alvarez-Salvado, E., Pallares, V. G., Moreno, A. & Canals, S., Functional MRI of long-term potentiation: imaging network plasticity, *Philos. Trans. R. Soc. Lond. B Biol. Sci.*, **369**, 20130152, (2014).

[23] Canals, S., Beyerlein, M., Murayama, Y. & Logothetis, N. K., Electric stimulation fMRI of the perforant pathway to the rat hippocampus, *Magn. Reson. Imaging*, **26**, 978-986, (2008).

[24] Canals, S., Beyerlein, M., Keller, A. L. Murayama, Y. & Logothetis, N. K. Magnetic resonance imaging of cortical connectivity in vivo, *Neuroimage*, **40**, 458-472, (2008).

[25] Jenkinson, M., Beckmann, C. F., Behrens, T. E., Woolrich, M. W. & Smith. S. M. FSL, *Neuroimage*, **62**, 782-790, (2012).

[26] Smith, S.M., Jenkinson, M., Woolrich, M.W., Beckmann, C.F., Behrens, T.E., Johansen-Berg, H., Bannister, P.R., De Luca, M., Drobnjak, I., Flitney, D.E. & Niazy, R.K., Advances in functional and structural MR image analysis and implementation as FSL, *Neuroimage*, **23**, 208-219, (2004).

[27] Cox, R. W., AFNI: software for analysis and visualization of functional magnetic resonance neuroimages, *Comput. Biomed. Res.*, **29**, 162-173, (1996).

[28] Cox, R. W., AFNI: What a long strange trip it has been, *Neuroimage*, **62**, 743-747, (2012).

[29] Jenkinson, M., Bannister, P., Brady, M. & Smith, S., Improved optimization for the robust and accurate linear registration and motion correction of brain images, *Neuroimage*, **17**, 825-841, (2002).

[30] Smith, S. M., Fast robust automated brain extraction, *Hum. Brain Mapp.*, **17**, 143-155, (2002).

[31] Schwarz, A.J., Danckaert, A., Reese, T., Gozzi, A., Paxinos, G., Watson, C., Merlo-Pich, E.V. & Bifone, A., A stereotaxic MRI template set for the rat brain with tissue class distribution maps and co-registered anatomical atlas: application to pharmacological MRI., *Neuroimage*, **32**, 538-550, (2006).

[32] Jenkinson, M. & Smith, S. A global optimisation method for robust affine registration of brain images, *Med. Image Anal.*, **5**, 143-156, (2001).

[33] Paxinos, G. & Watson, C. *The Rat Brain in Stereotaxic Coordinates*, (Academic Press, New York, 2007).

[34] Park, H. J. & Friston, K., Structural and functional brain networks: from connections to cognition, *Science*, **342**, 6158, (2013).

[35] Robinson, P. A., Sarkar, S., Pandejee, G. M. & Henderson, J. A., Determination of effective brain connectivity from functional connectivity with application to resting state connectivities, *Phys. Rev. E*, **90**, 012707, (2014).

[36] Deco, G. McIntosh, A. R., Shen, K., Hutchison, R. M., Menon, R. S., Everling, S., Hagmann, P. & Jirsa, V. K., Identification of optimal structural connectivity using functional connectivity and neural modeling, *J. Neurosci.*, **34**, 7910-7916, (2014).

[37] Robinson, P. A., Interrelating anatomical, effective, and functional brain connectivity using propagators and neural field theory, *Phys. Rev. E*, **85**, 011912, (2012).

[38] Sarkar, S., Chawla, S. & Xu, D., On inferring structural connectivity from brain functional-MRI data, arxiv.org/pdf/1502.06659, (2015).

[39] Hespanha, J. P., An efficient MatLab algorithm for graph partitioning, *University of California*, 1-8, (2004).

[40] Bullmore, E. & Sporns, O., Complex brain networks: graph theoretical analysis of structural and functional systems, *Nature Rev. Neurosci.*, **10**, 186-198, (2009).

[41] Friedman, J., Hastie, T. & Tibshirani, R., Sparse inverse covariance estimation with the graphical lasso, *Biostatistics*, **9**, 432-441, (2008).

[42] Erdös, P. & Rényi, A., On the evolution of random graphs, *Publ. Math. Inst. Hung. Acad. Sci*, **5**, 17-61, (1960).

[43] Bollobás, B. & Riordan, O., *Percolation*, (Cambridge University Press, Cambridge, 2006).

[44] Bollobás, B, *Random Graphs*, (Academic Press, London, 1985).

[45] Stauffer, D. & Aharony, A. , *Introduction To Percolation Theory: Revised* (Taylor & Francis, ed. 2, 1994).

[46] Gallos, L. K., Makse, H. A. & Sigman, M., A small world of weak ties provides optimal global integration of self-similar modules in functional brain networks, *Proc. Natl. Acad. Sci. USA*, **109**, 2825-2830, (2012).

[47] Granger, C. W. J., Investigating Causal Relations by Econometric Models and Cross-spectral Methods, *Econometrica*, **37**, 424-438, (1969).

[48] Roth, B. L., DREADDs for Neuroscientists. *Neuron* **17**, 683-694, (2016).

[49] Armbruster, B. N., Li, X., Pausch, M. H., Herlitze, S. & Roth, B. L., Evolving the lock to fit the key to create a family of G protein coupled receptors potently activated by an inert ligand, *Proc. Natl. Acad. Sci. USA*, **104**, 5163-5168, (2007).

[50] Alexander, G. M., Rogan, S. C., Abbas, A. I., Armbruster, B. N., Pei, Y., Allen, J. A., Nonneman, R. J., Hartmann, J., Moy, S. S., Nicolelis, M. A. & McNamara, J. O., Remote control of neuronal activity in transgenic mice expressing evolved G protein-coupled receptors, *Neuron*, **63**, 27-39, (2009).
